# Compost amendment in urban gardens: elemental and isotopic analysis of soils and vegetable tissues

**DOI:** 10.1007/s11356-024-34240-7

**Published:** 2024-07-10

**Authors:** Simone Trimmel, Stefan Wagner, Laura Feiner, Maria Feiner, Daniela Haluza, Rebecca Hood-Nowotny, Ulrike Pitha, Thomas Prohaska, Markus Puschenreiter, Philipp Spörl, Andrea Watzinger, Elisabeth Ziss, Johanna Irrgeher

**Affiliations:** 1 Department General, Analytical and Physical Chemistry, Montanuniversität Leoben, Leoben, Austria; 2 Department of Environmental Health, Center for Public Health, Medical University of Vienna, Vienna, Austria; 3 Department of Forest- and Soil Sciences, Institute of Soil Research (IBF), BOKU University, Vienna, Austria; 4 Department of Civil Engineering and Natural Hazards, Institute of Soil Bioengineering and Landscape Construction (IBLB), BOKU University, Vienna, Austria

**Keywords:** ICP-MS, Pb isotope ratios, Heavy metals, Soil remediation, Multielement analysis, Environmental monitoring, Urban agriculture, Food safety

## Abstract

Urban horticulture poses a sustainable form of food production, fosters community engagement and mitigates the impacts of climate change on cities. Yet, it can also be tied to health challenges related to soil contamination. This work builds on a previous study conducted on eleven urban gardens in the city of Vienna, Austria. Following the findings of elevated Pb levels in some soil and plant samples within that project, the present study investigates the elemental composition of soil and plants from two affected gardens 1 year after compost amendment. Inductively coupled plasma mass spectrometry (ICP-MS) analysis of skin, pulp and seeds of tomato fruits revealed minor variations in elemental composition which are unlikely to have an impact on food safety. In turn, a tendency of contaminant accumulation in root tips and leaves of radishes was found. Washing of lettuce led to a significant reduction in the contents of potentially toxic elements such as Be, Al, V, Ni, Ga and Tl, underscoring the significance of washing garden products before consumption. Furthermore, compost amendments led to promising results, with reduced Zn, Cd and Pb levels in radish bulbs. Pb isotope ratios in soil and spinach leaf samples taken in the previous study were assessed by multi-collector (MC-) ICP-MS to trace Pb uptake from soils into food. A direct linkage between the Pb isotopic signatures in soil and those in spinach leaves was observed, underscoring their effectiveness as tracers of Pb sources in the environment.

**Graphical Abstract:**

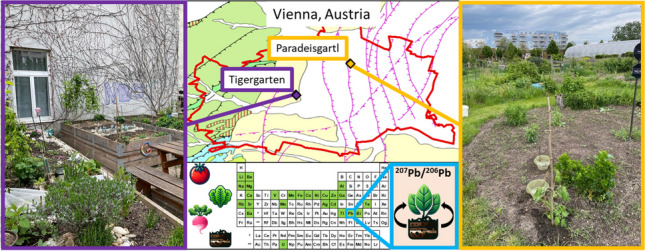

**Supplementary Information:**

The online version contains supplementary material available at 10.1007/s11356-024-34240-7.

## Introduction

Urban gardening has emerged as a widely adapted measure for sustainable food production in urban environments (Tomatis et al. 2023). A recent study found that with ideal use of space and resources, up to 82% of fresh vegetables consumed in Berlin could be produced within the city (De Simone et al. 2023), potentially reducing greenhouse gas emissions from food transport (Pradhan et al. 2020). Thus, urban gardening will be an essential component towards achieving the United Nation’s Sustainable Development Goals (SDGs) (Nicholls et al. 2020). However, urban gardening has also raised concerns related to the exposure to potentially toxic elements (PTEs) (Gaspéri et al. 2018; Islam et al. 2014; Rodríguez-Rodríguez et al. 2023; Säumel et al. 2012; Ziss et al. 2021). The contents of PTEs found in urban garden products are linked both to atmospheric deposition and to levels present in soil, which are impacted both by the soils’ different histories of land use (Clarke et al. 2015; Konwuruk et al. 2021) and present-day sources such as traffic and active industrial sites (Bassetti et al. 2023).

Various plant organs can interact differently with elements present in the soil, reflecting physiological variations in uptake, translocation and storage mechanisms (Hawkesford et al. 2023). As the primary interface with the soil, roots often exhibit elemental contents correlating most strongly with those in soil (Ray et al. 2021). They can sequester PTEs and prevent their upward mobility into above-ground plant tissues (Kafle et al. 2022), which, in turn, can also accumulate elements from atmospheric deposition. Examples are the uptake of nanoparticles via the stomata or diffusion of dissolved particles through aqueous pores of the cuticle (Uzu et al. 2010). Studies on iron (Fe), copper (Cu), zinc (Zn), cadmium (Cd) and lead (Pb) found that direct foliar uptake can be even more effective than via the roots (Žalud et al. 2012), likely due to the increased bioavailability of suspended and deposited dust fall (Preciado and Li 2006). Fruits and seeds generally have reduced contents of most elements (Bidar et al. 2020; Karahan et al. 2020). As they are located at the end of the nutrient transport chain, the uptake of non-essential elements is limited by mechanisms such as the root barrier (Cheng et al. 2020), cellular sequestration (Ulhassan et al. 2023), or preferential allocation to tissues like the stem (Galvis et al. 2023). Still, some elements such as Cd can hijack the transport pathways of essential nutrients (Sterckeman and Thomine 2020). Moreover, elemental accumulation patterns between different plant tissues depend on biological factors such as plant genotype (Arnold et al. 2010; Baker 1981) or symbiotic relationships with mycorrhizal fungi (Shukla et al. 2022).

To reduce the levels of PTEs which are particle-bound or loosely attached to plant surfaces, washing with water is an effective strategy (Augustsson et al. 2023a). The efficiency of washing varies based on the plant’s surface characteristics. For instance, smooth-skinned fruits like tomatoes shed contaminants more readily compared to rough or textured surfaces like strawberries or tomato leaves (Rodríguez-Rodríguez et al. 2023). Another important strategy to reduce PTE levels in food products is to apply soil amendments, which lower the bioavailability and hence the uptake of PTEs into plants (Yuan et al. 2023). Here, bioavailability refers to the immediate capacity of PTEs to traverse plant cell membranes, in contrast to bioaccessibility, which considers the elements’ potential availability subject to temporal and spatial constraints (Semple et al. 2004). Raised garden beds were found particularly effective to mitigate risks of contaminant exposure (Senderewich et al. 2024).

With the rise of modern technologies, awareness increased in the need for monitoring of technology-critical elements (TCEs), e.g. gallium (Ga) and thallium (Tl) (Cobelo-García et al. 2015; Romero-Freire et al. 2019). A study on the soil–plant transfer of selected TCEs to lettuce, carrot and chard found low uptake of Ga, niobium (Nb) and the rare-earth elements (REEs) compared to traditional contaminants, while the toxic element Tl was taken up more readily (Qvarforth et al. 2022). Still, despite the necessity of monitoring these emerging contaminants, the relevance of assessing health risks posed by traditional metal contaminants such as Pb remains unabated. Because of substantial fluctuations in the natural backgrounds of Pb in soil, coupled with its extensive industrial use in various applications (Friesl-Hanl et al. 2009), there exists a potential risk of elevated Pb levels in garden products.

To investigate the uptake of Pb from soil to plants, Pb isotope ratio analysis is a valuable approach. Pb possesses four stable isotopes: ^208^Pb, ^207^Pb, ^206^Pb and ^204^Pb. Pb isotopic abundances are site-dependent, which results from a varying combination of primordial and radiogenic components (Zhu et al. 2021). As a result, Pb isotope ratios are widely used for biogeochemical tracing in many fields of science, including geochronology, environmental studies and forensic science (Prohaska et al. 2000; Reese et al. 2020; Zhou et al. 2018). Analysing the isotopic ratios in soil can provide insights into historical pollution events (Prohaska et al. 2000) and the mobilisation of Pb into plants from different fractions of the soil (Hiller et al. 2022).

This study follows up the project ‘Heavy Metal City-Zen’ (HMCZ, https://heavymetalcityzen.com/, 10.55776/TCS74). In the project HMCZ, Zn, Cd and Pb contents were studied in soil, radish and spinach samples from eleven urban gardens in Vienna, Austria (Ziss et al. 2021). At two of these sites, Tigergarten and Paradeisgartl, elevated Pb mass fractions above the European food standard limits (European Commission 2023) and the Austrian agricultural guideline values (Austrian Standards 2017) were found in soil and/or plant samples. This has led to remediation measures involving mixing of the native soil with municipal compost. Based on this, further sampling and analyses were carried out in this study to assess the effectiveness of the conducted measures:

- Comprehensive elemental analysis: Based on a dataset encompassing 22 elements across 53 plant and 17 soil samples, a granular, plant organ-specific view of elemental distributions is provided. Patterns, variations, and potential risks associated with elemental uptake are investigated. A particular emphasis is placed on understanding the impact of washing on consumer exposure and on the investigation of the effectiveness of soil remediation efforts.
- Pb isotope ratio analysis: Given the potential health hazards associated with Pb and its pervasive presence in urban spheres, this study investigates ^207^Pb/^206^Pb isotope ratios using MC-ICP-MS to investigate Pb uptake from urban soils into food products. Though challenges exist, the endeavour provides an opportunity to refine methodologies and understand Pb behaviour in urban gardens.

## Materials and methods

Details about reagents and laboratory conditions as well as the applied calibration standards and certified reference materials (CRMs) are given in the supplementary information (SI) – 1.

### Sampling

**Fig. 1:**
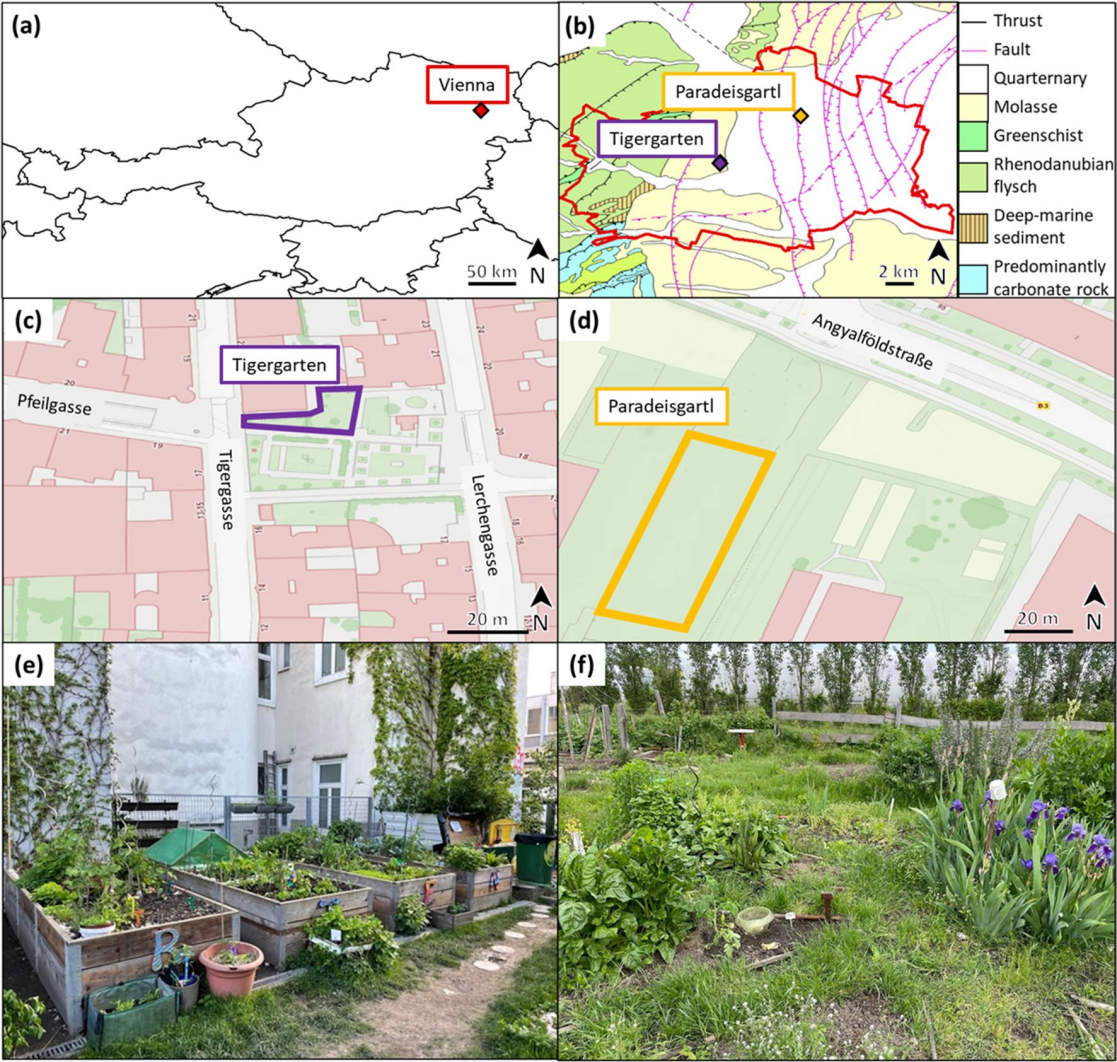
**a** Location of Vienna within Austria. **b** Location of the sampling sites in Vienna with lithography. **c** Overview of the Tigergarten location. **d** Overview of the Paradeisgartl location. **e** Picture from Tigergarten. **f** Picture from Paradeisgartl. The maps were created with ArcMap 10.8.1, © 2020 Esri

The sampled gardens were ‘Tigergarten’ (https://gartenpolylog.org/index.php/gartenprojekt/tigergarten), located at Pfeilgasse 19, 1080 Vienna (48.20887° (N), 16.34468° (E)), and ‘Paradeisgartl’ (https://gartenpolylog.org/en/node/895), situated at Angyalföldstraße/Hans-Czermak-Gasse, 1210 Vienna (48.25445° (N), 16.42472° (E)). A map of these locations is given in Fig. 1a–d.

As mentioned above, these sites were chosen due to elevated Pb levels in some soil and/or plant samples analysed in the project HMCZ. Austrian Standards sets the guideline value for acceptable Pb levels in agricultural soils at 100 mg kg^−1^ (Austrian Standards 2017). For agricultural products, the maximum permissible Pb levels are set by the European Commission (EC) at 300 µg kg^−1^ for leafy vegetables and at 100 µg kg^−1^ for root vegetables, both for wet weight (w.w.) (European Commission 2023). While the Pb mass fractions of up to 64 mg kg^−1^ in Tigergarten soil remained below the Austrian guideline, spinach leaves from the same location exceeded the limit value with up to 460 µg kg^−1^ (w.w.). In Paradeisgartl soil, Pb mass fractions reached up to 160 mg kg^−1^, surpassing the Austrian guideline. Furthermore, both spinach leaf and radish samples exceeded the EC limit values with 730 µg kg^−1^ (w.w.) and 230 µg kg^−1^ Pb (w.w.), respectively (Ziss et al. 2021).

As shown in Fig. 1e, the vegetable beds in Tigergarten are raised beds enclosed by wooden frames. These beds are filled with local soil mixed with compost and do not reflect the natural lithological background only. In turn, in-ground beds were used in Paradeisgartl (Fig. 1f), in which native soil is mixed with compost. In this case, the lithography of the region (see Fig. 1b) is expected to have affected elemental levels in the samples to a larger extent.

The sampling campaign in June 2020 involved sampling of spinach leaves (*Spinaca oleracea L. ‘*Butterfly’), radish bulbs and leaves (*Raphanus sativus L.* ‘Topsi’) and soil in both Tigergarten and Paradeisgartl. Soils consisting of a compost mixture of the gardeners’ choice were taken directly from the beds. Control soils were taken from the gardens’ neighbourhoods or adjacent to the beds. The soil samples were taken as composite samples from three to five subsamples of the top 10 cm within a radius of 2 to 4 m. The plants were grown for 5–8 weeks in buried pots either in amended soil or control soil. Further information can be found elsewhere (Ziss et al. 2021).

At the sampling campaign in July 2021, cherry tomato fruits (*Solanum lycopersicum* ‘Justens Zuckertomate’), radish bulbs and leaves (*Raphanus sativus L. ‘*Topsi*’*), lettuce leaves (*Lactuca sativa var. crispa* ‘Lollo Rossa’) and soil were taken in Paradeisgartl. In Tigergarten, only cherry tomato fruits and soil samples were available. Soil samples consisting of soil mixed with municipal compost were taken at two different depths (0 cm and 20 cm) in the beds next to the sampled plants. Control soil samples were taken at three different depths (0 cm, 20 cm and 30 cm) at a fallow spot in the back of the garden.

### Sample preparation

In the present study, soil extraction was performed with ammonium nitrate (NH_4_NO_3_) and aqua regia. The extraction protocols are based on the Austrian standards for soil analyses ÖNORM L 1094–1 and ÖNORM L 1085, respectively (Austrian Standards 2009, 2016). These extraction protocols were chosen to assess both the exchangeable and the pseudo-total pool size of elements present in the soil (de Vries et al. 2005; Stuanes et al. 1984). An overview of the samples taken at Tigergarten and Paradeisgartl in 2020 and 2021 is given in Table 1.

**Table 1:**
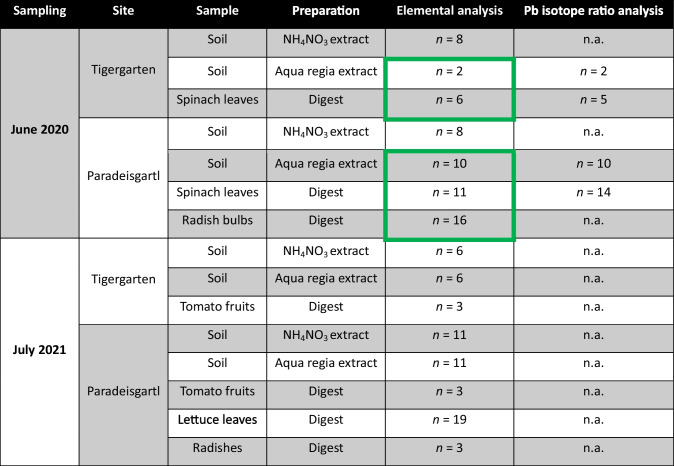
Samples taken at Tigergarten and Paradeisgartl in 2020 and 2021 with n.a. = not analysed. The analyses in the green frames were performed in the course of the project HMCZ (Ziss et al. 2021)

A different number of soil samples was extracted with NH_4_NO_3_ and aqua regia, and also the number of samples subjected to elemental versus isotopic analysis differed for the samples taken in June 2020 during the HMCZ project. This discrepancy occurred because these steps took place at different times and as part of different projects. The large sample set acquired in HMCZ necessitated selective processing in some instances, resulting in some samples not being analysed. Thereby, the number of samples analysed per garden was adjusted to reflect the size of the garden. As Tigergarten is much smaller than Paradeisgartl, some samples from Tigergarten were not analysed in HMCZ. Conversely, for Paradeisgartl, the smaller number of NH_4_NO_3_ extracts was due to only 8 samples having retained unprocessed soil material available.

The samples taken in June 2020 were processed and analysed for their mass fractions of Zn, Cd and Pb at the University of Natural Resources and Life Sciences, Vienna (BOKU) (Ziss et al. 2021). The aqua regia extracted soil samples and the digested spinach samples including CRMs and blanks were provided for MC-ICP-MS analysis of Pb isotope ratios. In addition, the air-dried and sieved soil samples were provided for NH_4_NO_3_ extraction.

The samples taken in July 2021 were processed and analysed for their mass fractions of beryllium (Be), sodium (Na), magnesium (Mg), aluminium (Al), calcium (Ca), vanadium (V), manganese (Mn), Fe, cobalt (Co), nickel (Ni), Cu, Zn, Ga, rubidium (Rb), strontium (Sr), molybdenum (Mo), silver (Ag), Cd, barium (Ba), Tl, Pb and U at Montanuniversität Leoben (MUL). Tomato fruits and radish bulbs were cut after washing using a ceramic knife on a plastic cutting board to avoid metal contamination. Skin, seeds and pulp of tomato fruits, as well as skin, roots and peeled bulbs of radishes were collected separately and freeze-dried using a ZS10N freeze drier (Drawell Scientific, China). The lettuce leaves were divided into two parts, one of which was washed thoroughly with reverse osmosis water, while the other part was left unwashed. Lettuce and radish leaf samples were oven-dried at 40 °C.

Microwave-assisted digestion of plant samples was conducted based on a previously published method (Trimmel et al. 2023). Briefly, 0.2 g of the plant tissues were digested along with the CRMs SRM1547 Peach Leaves, ZC73036a Green Tea or GBW10015 Spinach Leaves with 5 mL of HNO_3_ (*w* = 65%), 1 mL of H_2_O_2_ (*w* = 30%) and 0.1 mL of HBF_4_ (*w* = 38%) at 200 °C for 15 min using a Multiwave Pro microwave oven (Anton Paar, Austria). The digests were filled to 20 mL with ultra-pure water.

Soil samples were air-dried, sieved to a grain size smaller than 2 mm and stored at room temperature in plastic bags. For NH_4_NO_3_ extraction, 20 g of each sample were extracted with 50 mL of NH_4_NO_3_ solution (*c* = 1 mol L^−1^) by shaking for 2 h in a horizontal shaker at a frequency of 160 rpm. The shaken samples were then filtered and acidified with HNO_3_ (*w* = 65%) to a final HNO_3_ mass fraction of *w* = 2%. 10 g ± 3.5 g of each sample were finely ground in a ball mill MM400 (Retsch, Germany). For aqua regia extraction, 0.5 g of the ground samples were extracted with 3.75 mL of HCl (*w* = 32%) and 1.25 mL of HNO_3_ (*w* = 65%), again in a Multiwave Pro microwave oven. To avoid extensive formation of foam, the samples were left standing at room temperature for 30 min after addition of the chemicals. The microwave programme consisted of a 30 min temperature ramp step to 60 °C, 30 min hold, followed by another 30 min temperature ramp step to 135 °C and 60 °C hold. The extracts were filled to 50 mL with ultra-pure water and filtrated (0.45 µm).

To assess the mass fractions in whole tomato fruits and unpeeled radish bulbs based on the mass fractions in the individual parts, three tomatoes and five radishes were purchased from a local grocery store. After the products were washed with tap water in a household kitchen, their wet weight (w.w.) was determined. The fruits and bulbs were then divided into the individual parts and dried in the oven overnight (~ 8 h) at 90 °C to determine the moisture content. The dry weight (d.w.) was determined on an analytical balance in the laboratory.

### Multielement analysis

Plant digests were diluted 1:10 with ultra-pure water to achieve acidity corresponding to *w* = 2% HNO_3_. NH_4_NO_3_ soil extracts were diluted 1:50 with HNO_3_ (*w* = 2%). Aqua regia soil extracts were diluted 1:100 with HNO_3_ (*w* = 2%). Multielement ICP-MS analyses were performed using a NexION 2000 ICP-MS instrument (PerkinElmer, USA). Potential carry-over effects were monitored with HNO_3_ analysis blanks (*w* = 2%) which were measured after every four samples. Quality control (QC) solutions were measured after every ten samples. Indium (In) was used as internal normalisation standard to correct for matrix effects and instrumental drift throughout the analysis. After each sample, the system was rinsed with HNO_3_ (*w* = 3%). Detailed information about the instrumental parameters can be found in the SI – 1 (Table S4).

### Pb isotope ratio analysis

Isotope ratio analysis of Pb in aqua regia soil extracts (*n* = 12) and spinach leaf digests (*n* = 19) was performed. These samples were previously analysed for their elemental mass fractions in the project HMCZ in 2020. Details about soil and plant sampling, sample processing for multielement analysis, and multielement data are provided elsewhere (Ziss et al. 2021).

For matrix separation, an automated low-pressure ion exchange chromatography system (prepFAST-MC, Elemental Scientific, USA) equipped with a 3 mL bed column filled with DGA resin (TrisKem International, Bruz, France) was used based on a previously published approach (Zimmermann et al. 2019). Before column loading, the samples were diluted to obtain acidity corresponding to *c* = 2 mol L^−1^ HNO_3_. For spinach digests, this involved 1:1.05 dilution with HNO_3_ (*w* = 65%), and for aqua regia soil extracts, this involved 1:33.8 dilution with HNO_3_ (*w* = 2%) and further acidification with HNO_3_ (*w* = 65%). Details about the applied matrix separation steps are given in the SI – 1 (Table S5).

To check quantitative Pb recovery and effective separation from matrix elements, Pb eluates were screened using ICP-MS as described in the “Multielement analysis” section. For this, each eluate sample (*c* = 5 mol L^−1^ HNO_3_) was diluted 1:15 with ultra-pure water. QC solutions were diluted with ultra-pure water to obtain acidity corresponding to *w* = 2% HNO_3_ and screened along with the eluates. The separated Pb eluates were transferred to PFA vessels and placed on a hot plate at 120 − 140 °C until only a small droplet was left, which was then re-dissolved in 2–4 mL of HNO_3_ (*w* = 2%). Depending on the Pb mass fractions, the samples were further diluted with HNO_3_ (*w* = 2%) to obtain Pb mass fractions in measurement solutions of *w* = 25 ng g^−1^, *w* = 20 ng g^−1^ or *w* = 10 ng g^−1^.

Isotope ratio analysis was carried out with a Nu Plasma HR (NP048, Nu Instruments, UK) MC-ICP-MS instrument equipped with an Aridus II (Teledyne CETAC, USA) desolvating nebuliser system. The collector design of the MC-ICP-MS allowed for simultaneous recording of all Pb stable isotopes including ^204^Pb, ^206^Pb, ^207^Pb and ^208^Pb, as well as ^203^Tl and ^205^Tl for standard sample bracketing (SSB) correction and ^202^Hg for possible interference correction of Hg on *m*/*z* = 204. Mass fractions of Pb in samples and SRM981 SSB-standards were matched within ± 10% to enable external intra-elemental instrumental isotopic fractionation (IIF) correction (Prohaska et al. 2014). Moreover, Pb samples and SRM981 SSB-standards were spiked with a Tl solution, and raw intensities of ^205^Tl and ^208^Pb were matched within ± 10% to enable internal inter-elemental IIF correction (Prohaska et al. 2014). Detailed information about the instrumental parameters can be found in the SI – 1 (Table S6).

### Data processing

Multielement ICP-MS data was processed using the Syngistix software version 2 (PerkinElmer, USA). Blank correction was performed using procedural blanks. The standard deviation (*s*) of the procedural blanks was used to calculate limits of detection (*x*_L_ = 3 × *s*) and quantification (*x*_Q_ = 10 × *s*). Data evaluation was performed using Excel version 2108 (Microsoft, USA).

Isotope ratios of Pb in soil and spinach samples are reported using the delta (*δ*) notation (in ‰), where the isotope ratio is expressed relative to an isotopic reference standard (NIST SRM981) according to Eq. (1).

1 $${{\delta }_{\text{standard}}({}^{i}\text{Pb}/{}^{j}\text{Pb})}=\frac{{R}_{\text{sample},\text{ corrected}}}{{R}_{\text{standard},\text{ mean}}}-1$$

^*i*^Pb:Heavier isotope *i* of Pb (i.e. ^208^Pb, ^207^Pb or ^206^Pb).

^*j*^Pb:Lighter isotope *j* of Pb (i.e. ^207^Pb, ^206^Pb or ^204^Pb).

*R*_sample, corrected_:Isotope ratio of sample after blank and IIF correction.

*R*_standard, mean_:Isotope ratio of reference standard measured before and after sample.

The isotopic differences of Pb between soil and spinach samples are reported using the capital delta (*∆*) notation according to Eq. (2).

2 $$\Delta {({}^{i}\text{Pb}/{}^{j}\text{Pb})}_{\text{soil}/\text{plant}}={\delta }_{\text{standard}}{({}^{i}\text{Pb}/{}^{j}\text{Pb})}_{\text{soil}}-{\delta }_{\text{standard}}{({}^{i}\text{Pb}/{}^{j}\text{Pb})}_{\text{plant}}$$

Further details on multielemental and Pb isotopic data processing can be found in the SI – 1.

### Statistical analysis

SPSS Statistics version 28 (IBM, USA) was used for one- and two-way repeated measures analyses of variances (ANOVAs) and Tukey’s post hoc tests. Excel version 2108 (Microsoft, USA) was used for two-sided two-sample *t*-tests. In all statistical tests, *p* values below 0.05 were regarded as significant. Outliers were not removed considering the large natural variation in elemental mass fractions and the small sample size.

### Writing

The generative artificial intelligence (AI) technology ChatGPT (GPT-4, OpenAI, USA) was used in order to improve language and readability.

## Results and discussion

Detailed analytical results including validation for multielement and Pb isotope ratio analyses, *p* values of statistically significant observations, as well as *x*_L_ and *x*_Q_ are given in the SI—2.

### Cherry tomato fruits—elemental distribution

The mass fractions of Na, Mg, Al, Ca, Mn, Cu, Zn, Rb, Sr, Mo, Cd and Ba in the pulp, seeds and skin of tomato fruits sampled in Tigergarten and Paradeisgartl, as well as the standard deviation of the replicates are given in the SI – 2 (Table F1). Mass fractions of Be, Fe, Co, Ni, Ga, Ag, Tl, Pb and U were < *x*_Q_.

Two-way repeated measures ANOVAs were conducted to investigate the significance of the difference in elemental mass fractions between the fruit parts and the two gardens. While the mass fractions of Na, Mg, Rb, Sr, Cd and Ba were significantly higher in samples from Paradeisgartl than in those from Tigergarten, these differences are likely to result from different histories of the substrate and varying cultivation conditions. As mentioned in the “Sampling” section, in Paradeisgartl, vegetables were cultivated in in-ground beds in native soil mixed with compost. Conversely, in Tigergarten, the crops were grown in raised beds.

**Fig. 2:**
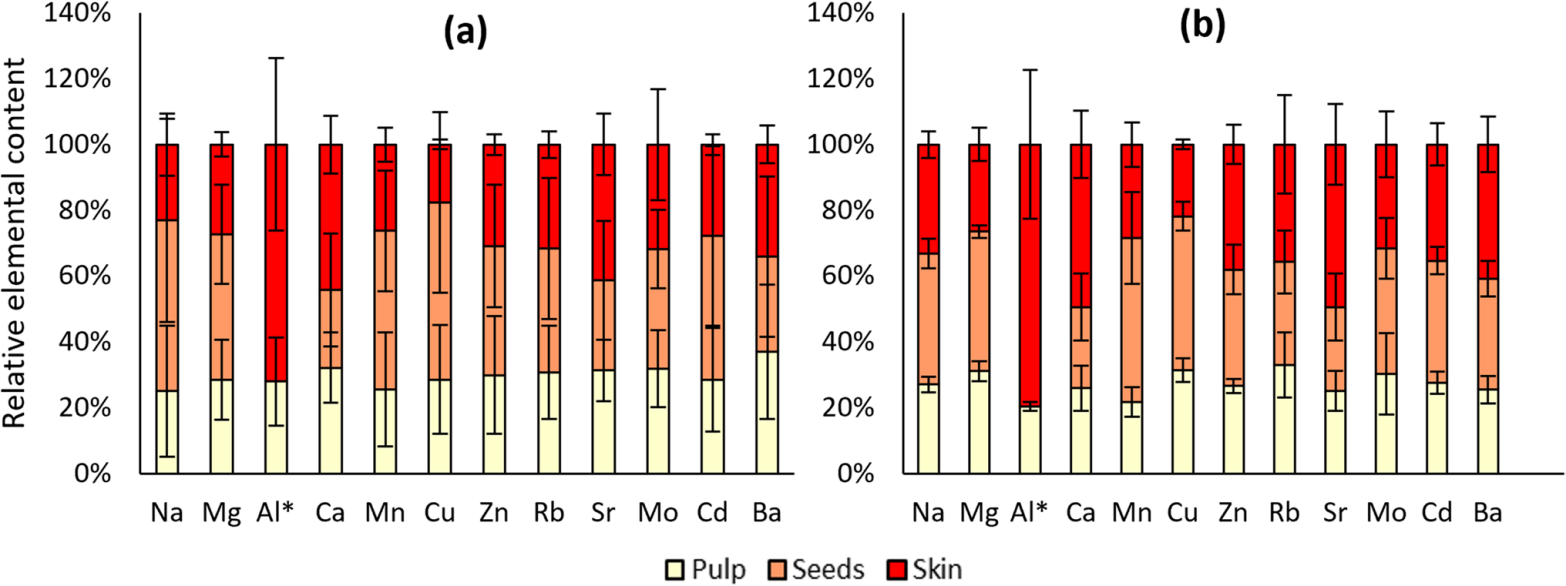
Relative elemental contents in different parts of tomato fruits sampled in **a** Tigergarten and **b** Paradeisgartl. The error bars indicate the standard deviation of the replicates (*n* = 3). *Al contents in tomato seeds were below *x*_Q_

The stacked column charts in Fig. 2 show the relative prevalence of the analytes between the fruit tissues. In the samples from both gardens, Mn and Cu were significantly highest in seeds. In addition, in Paradeisgartl samples, mass fractions found in the seeds were significantly higher than in the pulp in the case of Na (by 47%), and 55% higher compared to the skin in the case of Mg. Al, Ca and Sr mass fractions were significantly highest in the skin of the fruits. Particularly high levels of Al in the skin compared to the pulp were found.

The means of the dry masses of pulp (2.8 g), seeds (0.65 g) and skin (0.64 g) of a single cherry tomato fruit (*n* = 3) were assessed experimentally to evaluate possible consumer consequences of the presented findings, taking into account the actual proportions of the fruit parts. In addition, they were used to estimate the mass fractions for the whole fruits. These estimates are in accordance with mass fractions reported in literature for tomato fruits (Gundersen et al. 2001; Kovačič et al. 2023). The detailed comparison can be found in the SI—2 (Table F1).

To compare the obtained estimates with the limit values provided by the EC, they were converted to mass fractions in wet tissue based on the determined moisture content (*w* = 97%). The obtained results were all far below the limit values for the two elements commonly of concern Cd and Pb: Cd is estimated as 0.54 ng g^−1^ ± 0.24 ng g^−1^ (*s*, *n* = 6) in fresh whole fruits, compared to a limit value of 20 ng g^−1^ (European Commission 2023). As mentioned above, results for Pb were mostly below *x*_Q_, which corresponds to 0.15 ng g^−1^ in wet tissue. This is more than two orders of magnitude below the limit value of 50 ng g^−1^ (European Commission 2023).

Despite the obtained results remaining far below safety limits, the elemental distribution between fruit parts might come into play as an important factor to be considered for products made from concentrate or fruits grown on contaminated soil. The lowest mass fractions of most elements were found in the pulp. While the observed differences in elemental contents between the fruit parts are likely not large enough to be of concern to consumers, it also needs to be considered that the analyte mass fractions measured in the present study do not reflect human bioavailability, which can vary between plant tissues due to different compositions of the cell walls (Holland et al. 2020). Considering that the pulp makes up the largest part of a tomato fruit with about 68% of its dry mass, the largest share of the absolute content of the 12 investigated elements present in a tomato is found in the pulp (63% ± 3%).

Thus, processing steps involving the removal of seeds and skins of tomatoes such as straining cannot be considered effective measures to reduce levels of elements of concern. However, the elemental distribution between fruit parts might change depending on mobile levels present in soil, especially in contaminated areas (Chen et al. 2023). In addition, different plant species can show varying behaviour in terms of how strongly elements are accumulated in different fruit parts, which could make removal of seeds and skin beneficial in some cases.

### Lettuce leaves—effectiveness of washing for removal of surface-bound elements

**Fig. 3:**
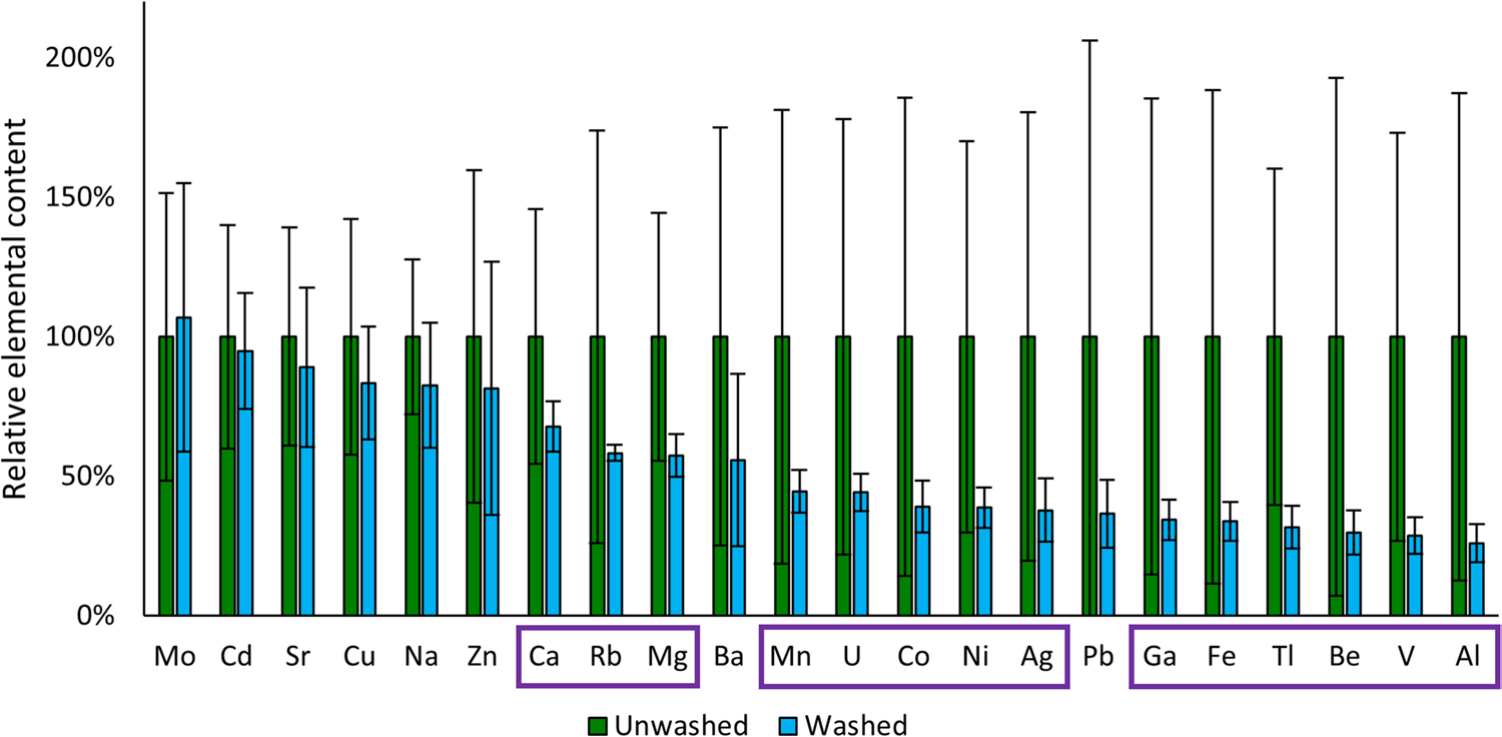
Comparison of relative elemental contents in unwashed (*n* = 6) and washed lettuce leaves (*n* = 13). Elements where statistically significant differences were found are indicated with a lilac rectangle

The mass fractions of Be, Na, Mg, Al, Ca, V, Mn, Fe, Co, Ni, Cu, Zn, Ga, Rb, Sr, Mo, Ag, Cd, Ba, Tl, Pb and U in washed and unwashed lettuce leaves sampled in Paradeisgartl are given in the SI – 2 (Table F2). Figure 3 shows the wash effect for each element along with the standard deviation of replicate samples. The data is sorted in ascending order by the wash effect, which was calculated by subtraction of the quotient of the mass fractions in washed against unwashed leaves. In Table F2, the wash effect values are provided with the propagated error from the respective standard deviation of the mass fractions with a coverage factor of *k* = 2 (see equation (S1) in SI – 1). The difference between the elemental mass fractions in unwashed and washed leaves was tested for statistical significance using a two-sided two-sample *t*-test with unequal variances.

Significantly lower mass fractions of Be, Mg, Al, Ca, V, Mn, Fe, Co, Ni, Ga, Rb, Ag, Tl and U were found in washed compared to unwashed samples. The elemental mass fractions found in unwashed leaves varied strongly, especially for elements taken up less readily into the plants, with RSDs of replicate samples ranging from *σ* = 28% for Na to *σ* = 106% for Pb. As the local adhesion of soil and dust to lettuce leaves is a highly variable parameter, this spread is expected. In turn, mass fractions found in washed leaves show a much narrower distribution, reflecting actual elemental uptake into the plants.

A comparison with literature data is given in the SI – 2, Table F2 (Augustsson et al. 2023b; Intawongse and Dean 2008). Overall, the findings are in good agreement with literature with the exception of Al. Al mass fractions are higher in the samples analysed in the present work by about one order of magnitude compared to a study (Augustsson et al. 2023b) conducted on four different types of leafy vegetables (chard, lettuce, parsley and kale). In both studies, washing did not lead to a significant change in the mass fractions of Mo, Cd, Cu, Na, Zn and Ba, while it did so for U, Co, Ni, Ga, Fe, Tl, Be, V and Al. In contrast, a significant reduction of Ca, Rb, Mg and Mn was found in the present work as opposed to the cited study, whereas the latter discovered a significant reduction of Pb mass fractions caused by washing.

These disparities are suspected to stem from an interlinkage of factors, including variations in soil compositions with different bioavailable or bioaccessible element fractions and plant-specific element uptake. Another likely influential factor is the quantity and composition of atmospheric particulate deposition, which varies strongly based on location, season and weather conditions. Consequently, when considering the previous study’s outcomes, it is possible to identify a common pattern of preferentially removing elements that are not physiologically relevant to plants during the washing process.

The presented findings underscore the importance of washing vegetables before analysis to increase sample homogeneity. Washing as a preparatory step allows for a more accurate assessment of elements within the plant tissue rather than external variables like soil or atmospheric deposition. Further, washing ensures that the analysis reflects the actual intake by consumers. In addition, the more consistent elemental distribution in washed samples simplifies comparisons across different studies. In the present study, lettuce leaves were chosen for the assessment of the impact of household washing on elemental mass fractions because they are growing directly above ground and are thus particularly exposed to direct contamination from soil. The leaves pose a large surface on which particles can adhere and are consumed directly without a possibility to remove the peel. As leafy vegetables, they tend to accumulate higher contents of Pb than bulbs or, even more so, fruits (Hadayat et al. 2018; Sultana et al. 2022).

Converted to fresh weight assuming a moisture content of 96% in lettuce leaves based on a previous study (Wang et al. 2021), Pb mass fractions of 440 ng g^−1^ and 160 ng g^−1^ and Cd mass fractions of 13 ng g^−1^ and 12 ng g^−1^ are estimated for unwashed and washed leaves, respectively. With EC limit values of 300 ng g^−1^ for Pb and 100 ng g^−1^ for Cd in leafy vegetables (European Commission 2023), washing lettuce before consumption has a negligible impact on Cd intake, but is highly advisable to avoid Pb exposure and eventually chronic Pb intoxication. This finding is particularly relevant in the light of a recent study reporting on Pb exposure from soil and dust ingestion contributing significantly to children’s blood levels of Pb in the USA (Mushak 2011; Zartarian et al. 2023).

### Radishes—elemental distribution

The mass fractions of Be, Na, Mg, Al, Ca, V, Mn, Fe, Co, Cu, Zn, Ga, Rb, Sr, Mo, Ag, Cd, Ba, Tl, Pb and U found in the radish leaves and the different parts of the radish bulbs (peeled bulb, root tip and skin) sampled in Paradeisgartl are given in the SI – 2 (Table F3) along with the standard deviation of the replicates. In addition, estimated mass fractions for the unpeeled bulbs are given with the errors propagated from the standard deviation of the individual parts with a coverage factor of *k* = 2 (see equation (S3) in SI – 1). The estimates are based on the means of the experimentally determined dry masses of the skin (0.21 g) and the peeled bulb (0.99 g) of a single radish (*n* = 5). Root tips were not considered in this calculation because they are usually not consumed.

**Fig. 4:**
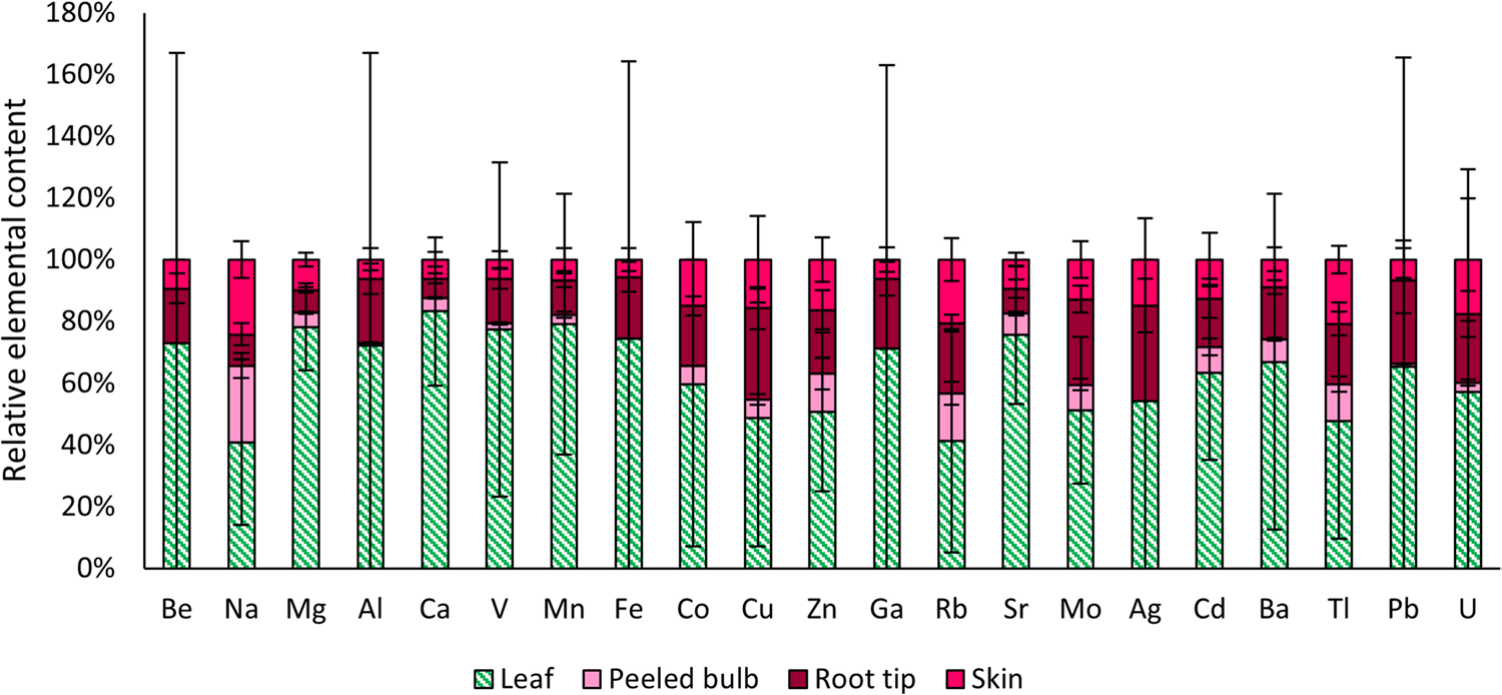
Relative elemental contents in different parts of radishes sampled in Paradeisgartl. Error bars indicate standard deviation of the replicates (*n* = 3). Be, Fe, Zn and Mo contents in peeled radish bulbs were below *x*_Q_

Differences in elemental mass fractions between radish leaves, peeled bulbs, root tips and skin were tested for statistical significance with 1-way repeated measures ANOVAs. Figure 4 visualises the relative elemental contents in different parts of the radish plants. While the contents of all elements were highest in the leaves, this is significant only for Mg, Ca, Mn, Sr and Cd. Mass fractions of Zn were significantly higher in leaves compared to the peeled bulbs, and Mo was significantly higher in leaves than in the peeled bulbs and the skin.

Assuming a moisture content of 87.5% in radish leaves based on the results of a previous study (Prasad 2015), the Pb content can be estimated to be 890 ng g^−1^ in fresh leaves, exceeding the EC limit value of 300 ng g^−1^ for washed leafy vegetables (European Commission 2023). Taking into account that a highly variable mean Pb content reduction by washing (63% ± 110%, see the “Lettuce leaves—effectiveness of washing for removal of surface-bound elements” section) was found in the experiments performed on lettuce leaves in this study, the limit value may be exceeded in some replicates even if they were washed. In contrast, the Cd mass fraction in fresh radish leaves can be estimated in the same way to 39 ng g^−1^, which remains below the EC limit of 100 ng g^−1^ despite not washing the leaves (European Commission 2023).

Most of the investigated elements tend to be lowest in the peeled bulb. However, due to the high variability between the samples which is further increased by taking the leaves into account, the variations within the radish bulbs were not found to be significant in the ANOVAs. Still, it can be seen from Fig. 4 that there is an indication of Al, V, Mn, Cu, Zn, Ga, Mo, Ag and Pb accumulating predominantly in the root tips, whereas Co, Cd, Tl and U mass fractions are elevated both in the root tips and the skin compared to the peeled bulbs. Thus, removal of the skin and in particular the root tips and secondary roots before consumption is advisable to reduce contaminant ingestion from radishes grown on soil with known elevated levels of toxic elements.

### Effectiveness of compost amendment

**Fig. 5:**
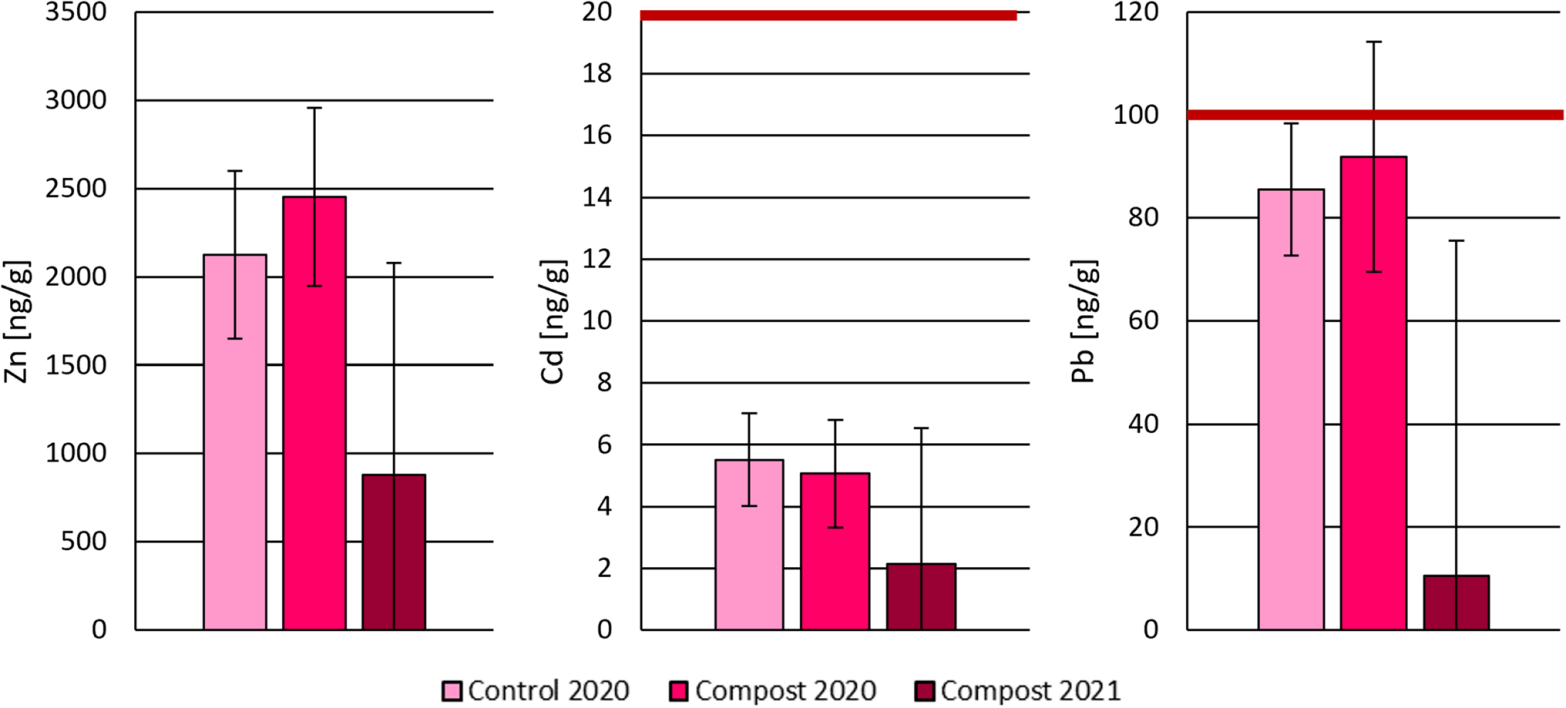
Zn, Cd and Pb mass fractions (converted to w.w.) of unpeeled radish bulbs grown in Paradeisgartl in 2020 (Ziss et al. 2021) on amended compost mixture (*n* = 4, light pink) and a control group of untreated soil (*n* = 6, medium pink), and estimates based on the results from 2021 for peeled bulbs and skin obtained in the present study (*n* = 3, dark pink). Red horizontal lines indicate the EC limit values (European Commission 2023) for Cd (20 ng g^−1^) and Pb (100 ng g.^−1^). Error bars indicate standard deviation of replicates or, in case of samples from 2021, the propagated error based on standard deviation of skin and peeled bulbs expanded with a coverage factor of *k* = 2

The estimated mass fractions of Zn, Cd and Pb in unpeeled radish bulbs were compared with the mass fractions obtained previously for radishes from Paradeisgartl (Ziss et al. 2021) to assess the impact of the application of externally produced compost mixtures. The bar charts in Fig. 5 show the results from both studies converted to fresh weight based on the experimentally determined moisture content of *w* = 95% in comparison with the EC limit values for Cd and Pb (European Commission 2023). For Zn, no threshold values are established.

In the case of all three analytes, lowest mass fractions were found in the most recent samples taken in the present study. The differences in mass fractions between the radishes grown in 2020 in the vegetable beds (Compost 2020) and those grown on soil from the garden’s neighbourhood or soil adjacent to the beds (Control 2020) were not statistically significant. In contrast, the radishes taken in 2021 had significantly lower elemental content in all observed cases except of the comparison with Cd content in radishes from the amendment group (*p* = 0.065).

**Fig. 6:**
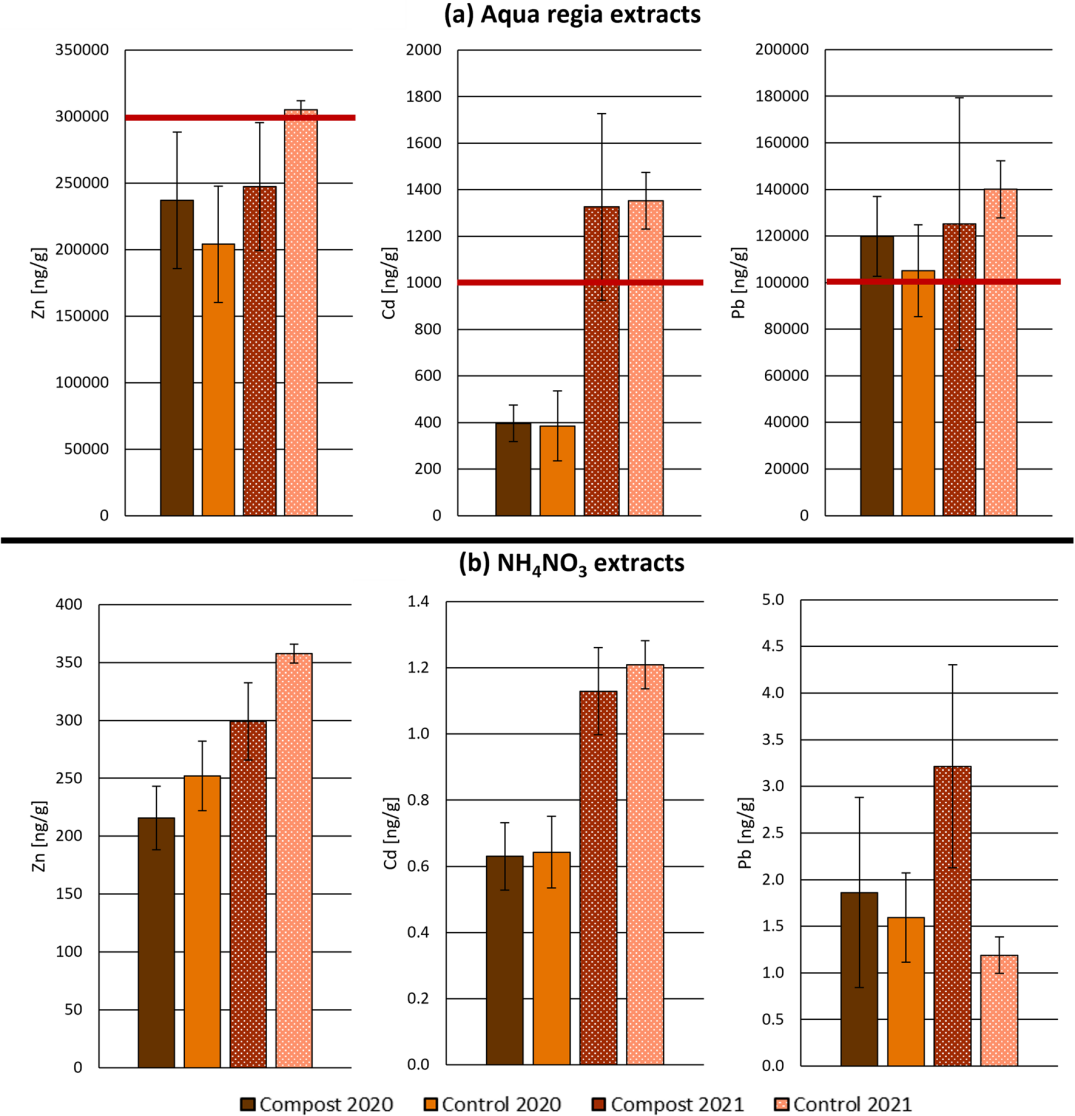
Zn, Cd and Pb mass fractions in **a** aqua regia and **b** NH_4_NO_3_ extracts of soil sampled in Paradeisgartl in 2020 (Ziss et al. 2021) from amended compost mixture (*n* = 4, dark brown) and a control group of untreated soil (*n* = 4, light brown), and extracts of soil sampled in 2021, from amended compost mixture (*n* = 8, medium brown) and a control group of untreated soil (*n* = 3, light pink). Red horizontal lines indicate the Austrian agricultural guideline values for Zn (300,000 ng g^−1^), Cd (1000 ng g^−1^) and Pb (100,000 ng g^−1^) in aqua regia extracts (Austrian Standards 2017). Guideline values in NH_4_NO_3_ extracts (40 ng g^−1^ for Cd and 300 ng g^−1^ for Pb (Austrian Standards 2014)) are not shown to preserve readability of the diagram. Error bars indicate standard deviation of replicates

The Cd content of the radish bulbs had not been of concern in the previous study either, but Pb mass fractions had exceeded the EC limit values in some cases. The strongly reduced Pb contents in the samples from 2021 remain far below the limit value. While this may indicate effectiveness of compost amendment for soil remediation, this observation is not reflected in the Zn, Cd and Pb contents in soil extracts, as shown in Fig. 6a, b. NH_4_NO_3_ extracts are applied to determine the exchangeable pool size as an estimate of the bioavailable fraction of trace elements in the soil, reflecting the elements that are more readily accessible to organisms (Austrian Standards 2014). In contrast, aqua regia soil extraction provides a comprehensive assessment of the operationally defined pseudo-total pool size of elements (Austrian Standards 2009, de Vries et al. 2005).

While the aqua regia extracts of 2020 soils exceeded only the Austrian agricultural guideline value of 100,000 ng g^−1^ Pb (Austrian Standards 2017), the samples from 2021 exceeded also the threshold of 1000 ng g^−1^ Cd. The Cd and Pb levels in NH_4_NO_3_ extracts, however, were far below the Austrian agricultural guideline values of 40 ng g^−1^ and 300 ng g^−1^, respectively (Austrian Standards 2014). Yet, Zn and in particular Cd mass fractions increased from 2020 to 2021, both in aqua regia and NH_4_NO_3_ extracts, and particularly in control soils. Considering that the control samples originate from parts of the garden unchanged since the initial sampling, this increase likely does not stem from the remediation actions. Instead, it may be due to the inherent soil variability appearing more prominently, as the 2021 samples were not composite (Li 2019).

The mismatch between the elemental content in radish bulbs and soil extracts points to complex interactions between soil composition and plant uptake. Plants possess tolerance mechanisms that effectively exclude harmful elements up to a certain threshold by the activation of cellular detoxification and sequestration (Ghuge et al. 2023). Thus, the relationship between levels present in soil and plant uptake is not linear. As the rhizosphere is the plants’ first line of defence against toxic elements (Podar and Maathuis 2022), the development of a healthier root system in a nutrient-rich compost-amended soil could have enabled the radishes to preferentially take up essential nutrients over toxic elements like Pb.

Furthermore, composition changes due to compost amendment may have affected Zn, Cd and Pb bioavailability or bioaccessibility by enhancing the soil’s capability to immobilise these elements. While the pseudo-total and exchangeable pool sizes of Zn, Cd and Pb might not have changed significantly in the Paradeisgartl vegetable beds, these do not necessarily indicate unchanged bioavailability or -accessibility. A more reliable measure is given by the rate at which the element is resupplied to the soil solution from which plants can absorb it (Tandy et al. 2011). This dynamic is affected by the soil pH, cation exchange capacity and presence of organic matter, which are altered by compost amendments. The compost itself can act as a sink for Zn, Cd and Pb, reducing their lability and resupply in soil, thereby affecting their bioavailability (Zeng et al. 2015).

For instance, a recent study indicated that organic amendments can enhance soil quality by increasing dissolved organic carbon, soil respiration and soil microbial carbon content, all of which contribute to a reduction in the bioavailability of heavy metals. Specifically, the combined application of organic materials such as biochar and compost has been observed to significantly reduce shoot Cd mass fractions in lettuce, while increasing the activity of catalase, an enzyme indicative of improved plant health (Yuan et al. 2023). Therefore, in addition to conventional soil extractions, future work should also apply dynamic extraction methods, such as the diffusive gradients in thin-films (DGT) technique, for a more comprehensive appreciation of the complex processes affecting contaminant lability and resupply dynamics in soil and associated changes in availability for plant uptake (Degryse et al. 2009; Smolders et al. 2020; Wagner et al. 2022; Zhang and Davison 2015).

Given that plant species vary in their uptake of potentially toxic elements, caution is advised regarding the food safety of crops from Paradeisgartl. Although the observed reduction in Zn, Cd and Pb within radish bulbs is encouraging, further research is needed to investigate the long-term implications of using compost for limiting the soil–plant transfer of potential contaminants in urban gardens.

### Pb isotope ratios

Pb isotope ratios were assessed to investigate whether the Pb in the plant samples can be directly linked to the aqua regia leachable fraction in soil. The Pb isotope ratios for soil and spinach leaf samples, expressed as *δ*-values, as well as the corresponding isotopic difference between soil and plant, expressed as *Δ*-values, are listed in the SI – 2 (Table G1-G3). A focus is placed on *δ*_SRM981_(^207^Pb/^206^Pb) values due to a combined effect of (a) the similar natural isotopic abundance (*A*) of ^207^Pb (*A* =  ~ 22.1%) and ^206^Pb (*A* =  ~ 24.1%) and hence improved analytical precision during MC-ICP-MS analysis, and (b) the small mass difference between ^207^ and ^206^Pb of only 0.5% and hence reduced potential for mass-dependent isotopic fractionation as compared to other Pb isotope ratios. No significant differences between *δ*-values obtained after IIF correction using external (SSB) or internal (Tl) calibration were observed with respect to the uncertainty (Table G1 and G2). Thus, only SSB-calibrated values are further discussed below. Elemental recoveries of Pb after automated matrix separation were fully quantitative with mean values of 101% ± 6% for soils and 107% ± 10% for plants (Table H1-H2 in the SI – 2).

The measured *δ*_SRM981_(^207^Pb/^206^Pb) values for both the soil and the spinach leaf samples from Tigergarten show a large spread (Table G1 and G2). In spinach grown on the compost-treated soil, a larger spread (expressed as *s*) of the mean *δ*_SRM981_(^207^Pb/^206^Pb) values (− 59‰ ± 17‰; *n* = 2) as compared to the control soil (− 67‰ ± 1‰; *n* = 3) was observed. This indicates that different sources of Pb are mobilised in the compost-treated soil. Still, the large spread of the *δ*_SRM981_(^207^Pb/^206^Pb) values in the Tigergarten soil requires a larger dataset for further interpretation.

With a *δ*_SRM981_(^207^Pb/^206^Pb) range of − 60.74 to − 68.90‰, the soil and spinach samples of Paradeisgartl exhibited a more homogeneous Pb isotopic signature than those of Tigergarten (Table G1 and G2). Here, two-sample two-sided *t*-tests with equal variances showed no significant difference between treated soil samples and the control soil (*p* = 0.4719), indicating no influence of compost on Pb isotope signatures in this soil. Between spinach samples grown on treated or control Paradeisgartl soil, no significant differences were observed either (*p* = 0.6871). It can be interpreted that the same sources of Pb in the soil are mobilised even though, as for Tigergarten, compost treatment resulted in a more variable Pb isotopic composition in spinach, as evidenced by the higher variability of *δ*_SRM981_(^207^Pb/^206^Pb) values in spinach grown on the compost-treated soil (− 65‰ ± 2‰; *n* = 7) as compared to the control soil (− 66‰ ± 1‰; *n* = 7). As can be seen in Fig. 6 (“Effectiveness of compost amendment” section), the aqua regia leachable Pb content is comparable between the two soil treatments, whereas the NH_4_NO_3_ leachable fraction (exchangeable pool size) is higher for the compost-treated soil. This indicates that different sources of Pb are mobilised in the compost-treated soil.

**Fig. 7:**
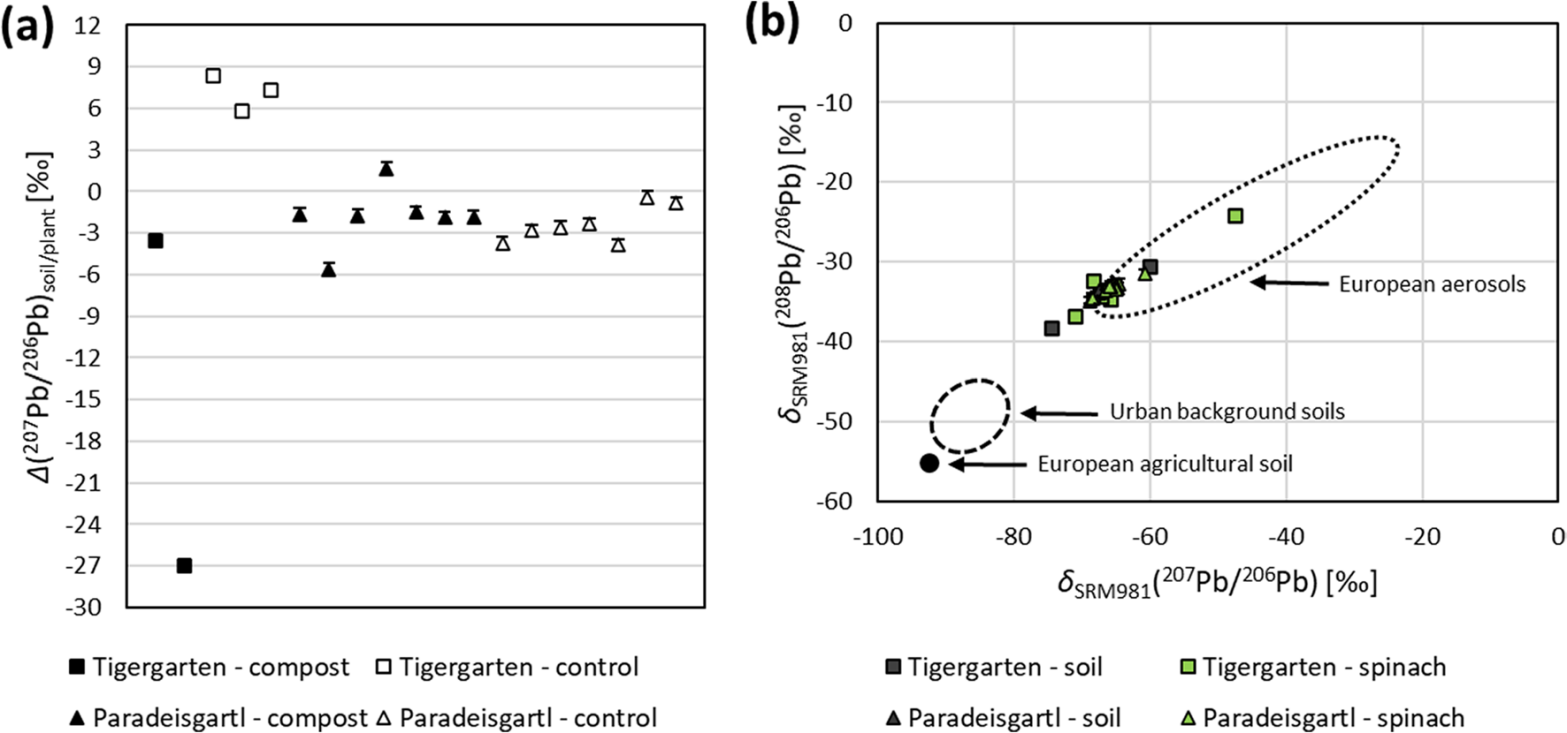
**a** *Δ*(^207^Pb/^206^Pb)_soil/plant_ values and **b δ**_SRM981_(^207^Pb/^206^Pb) values versus *δ*_SRM981_(^208^Pb/^206^Pb) values for soil samples and spinach leaf samples. References for European aerosols (Hiller et al. 2022), urban background soils that were taken at least 600 m away from roads (Hiller et al. 2020) and European agricultural soil (Reimann et al. 2012) are given based on literature values Error bars indicate *U* (*k* = 2). For Tigergarten samples, the error bars are smaller than the symbols

Figure 7a presents the *Δ*(^207^Pb/^206^Pb)_soil/plant_ values. In general, a *Δ*(^207^Pb/^206^Pb)_soil/plant_ value of zero or close to zero indicates that either no fractionation of Pb takes place during uptake or that the plant takes up Pb from a different source as compared to the aqua regia leachable fraction. Isotopic fractionation from soil to plants typically involves kinetic effects resulting in the preferred uptake of the lighter isotopes. This is because they are more easily released into the soil solution due to their lower molecular bond dissociation energy (Arnold et al. 2015; Chenery et al. 2012; Hindshaw et al. 2012; Ryan 2013; Schauble 2004). For example, soil–plant isotopic fractionation is reported for lighter elements in literature as *Δ*(^11^B/^10^B)_soil/plant_ =  − 0.2‰ (Xiao et al. 2022) or *Δ*(^65^Cu/^63^Cu)_soil/plant_ =  − 1‰ (Ryan 2013). Therefore, *Δ*(^207^Pb/^206^Pb)_soil/plant_ values could be expected to be slightly negative. However, as the relative mass difference between ^207^ and ^206^Pb of 0.5% is rather small, the extent to which soil–plant Pb isotopic fractionation can be expected to be minor.

The *Δ*(^207^Pb/^206^Pb)_soil/plant_ values close to zero in the Paradeisgartl data indicate a direct linkage between the Pb isotopic signature in the soil and that taken up in spinach leaves. In contrast, the Tigergarten compost samples show larger variations, suggesting Pb from different sources. The *Δ*(^207^Pb/^206^Pb)_soil/plant_ values in the Tigergarten control are more homogeneous. However, the significant difference in Pb isotope ratios between the spinach leaves and soil in the control samples indicates that Pb in the leaf samples originates from a specific part of the aqua regia leachable fraction, likely the one which is most readily mobilised. Therefore, it can be assumed that Pb in Tigergarten comes from multiple sources (pseudo-total pool size vs. exchangeable pool size), while Pb in the Paradeisgartl soil comes from a single source.

The three-isotope plot in Fig. 7b displays the *δ*_SRM981_(^207^Pb/^206^Pb) values against the *δ*_SRM981_(^208^Pb/^206^Pb) values both for spinach and soil samples from the two gardens. Literature data is given in the diagram to provide context: the European agricultural soil (Reimann et al. 2012) and the urban background soils, taken in the city of Bratislava in at least 600 m distance from roads (Hiller et al. 2020), serve as a proxy for uncontaminated soil with purely natural Pb sources, while the European aerosols (Hiller et al. 2022) represent anthropogenic impact. Comparing the samples from Tigergarten and Paradeisgartl with literature data suggests that the Pb isotopic composition of the sample set is primarily influenced by anthropogenic sources. Notably, one spinach sample from Tigergarten has an isotopic signature particularly different from that of the uncontaminated soils indicated in the diagram. This sample also exhibits a strongly negative *Δ*(^207^Pb/^206^Pb)_soil/plant_ value of − 27‰, as shown on the far left in Fig. 7a. Therefore, it appears that Pb in this sample predominantly stems from atmospheric deposition.

## Conclusion

The presented findings deepen the current understanding of the elemental dynamics in urban gardens with a focus on food safety measures and the efficacy of remediation strategies. Key findings include the effectiveness of washing in reducing surface-bound elements in lettuce leaves, particularly for Be, Al, V, Ga and Tl. Essential nutrients remained mostly intact after washing, which indicates that the nutritional value is not compromised. These findings emphasise the critical role of washing in ensuring food safety, highlighting the necessity for information campaigns targeting urban gardeners and the wider public to promote awareness and adoption of this practice.

The investigation of the distribution of elements within tomato fruit tissues revealed nuanced patterns which offer a deeper understanding of elemental accumulation. Yet, the differences in elemental mass fractions between the tissues are too small to point towards a significant enhancement in food safety through removal of seeds and skin. In radishes, elevated mass fractions of PTEs in the root tips and secondary roots as well as in the skin suggest that the removal of these parts can be beneficial, especially for vegetables grown in contaminated soils. The impact of compost amendments in reducing Zn, Cd and Pb in radish bulbs is a promising finding, highlighting the potential of soil remediation efforts in urban agriculture. This aligns with the goal of producing safe and nutritious food in urban settings.

The analysis of Pb isotope ratios offers insights into urban soil–plant dynamics. While the limited dataset in one garden does not allow definite conclusions, the consistency in Pb isotopic signatures between soil and spinach samples in another garden is a notable finding. It underscores the potential of using Pb isotopes as reliable tracers of Pb sources in agricultural environments. Measuring Pb isotope ratios along a soil depth profile of the garden sites and the analysis of several different plant species and additives such as compost mixtures could lead to a deepened understanding of soil–plant interactions and ultimately aid in the development of safer gardening practices and more effective soil remediation strategies.

Overall, this study contributes to the ongoing discourse on urban food safety, reinforcing the necessity for continuous monitoring and proactive management of soil health to simultaneously protect human health. The findings also open questions for future research, particularly concerning the refinement of methods for tracing of contamination. The insights gained in this work are not only relevant for urban gardeners, but also for policymakers, public health experts and environmental scientists aiming to foster sustainable and safe urban food production.

## Supplementary Information

Below is the link to the electronic supplementary material.

Supplementary file1 (PDF 304 KB)

Supplementary file2 (XLSX 120 KB)

## Data Availability

The authors declare that the data supporting the findings of this study are available within the paper and its Supplementary Information files. Should any raw data files be needed in another format, they are available from the corresponding author upon reasonable request.
