# Supplementary material for: Compost amendment in urban gardens: elemental and isotopic analysis of soils and vegetable tissues
Source: Environ Sci Pollut Res Int. 2024 Jul 10;31(34):47022–38. doi: 10.1007/s11356-024-34240-7 (PMC11512910; doi:10.1007/s11356-024-34240-7)
Supplement: Supplementary file 1 — Supplementary file1 (PDF 304 KB) [file 11356_2024_34240_MOESM1_ESM.pdf]

Supplementary Information – 1

Compost amendment in urban gardens: elemental and isotopic analysis of soils and vegetable tissues

Simone Trimmel[a], Stefan Wagner[a], Laura Feiner[a], Maria Feiner[a], Daniela Haluza[b], Rebecca Hood-Nowotny[c], Ulrike Pitha[d], Thomas Prohaska[a], Markus Puschenreiter[c], Philipp Spörl[d], Andrea Watzinger[c], Elisabeth Ziss[c], Johanna Irrgeher[a]*

[a]Montanuniversität Leoben, Department General, Analytical and Physical Chemistry, Chair of General and Analytical Chemistry, Austria

[b]Medical University of Vienna, Center for Public Health, Department of Environmental Health, Austria

[c]University of Natural Resources and Life Sciences, Vienna, Department of Forest- and Soil Sciences, Institute of Soil Research (IBF), Austria

[d]University of Natural Resources and Life Sciences, Vienna, Department of Civil Engineering and Natural Hazards, Institute of Soil Bioengineering and Landscape Construction (IBLB), Austria

*Correspondence: johanna.irrgeher@unileoben.ac.at

Table of contents

1. Reagents and laboratory conditions 2

2. Calibration standards and certified reference materials (CRMs) 2

3. Multielement analysis 5

4. Pb isotope ratio analysis 6

5. Data processing 7

5.1 Multielement analysis 7

5.2 Pb isotope ratio analysis 7

References 8

1. Reagents and laboratory conditions

Ultra-pure water was obtained from a Milli-Q IQ 7000 module (18.2 MΩ cm; Merck Millipore, Germany). Hydrochloric acid (HCl, *w* = 37 %, p.a. grade; Carl Roth GmbH, Germany) and nitric acid (HNO_3, *w* = 65 %, p.a. grade; Carl Roth GmbH, Germany) were further purified in perfluoralkoxy-polymer (PFA) sub-boiling units (DST−1000 and DST-4000, Savillex, USA). Sub-boiling reduced the HCl mass fraction to *w* = 32 %. Hydrogen peroxide solution (H_2O_2, *w* = 30 %; Merck KGaA, Germany) and tetrafluoroboric acid (HBF_4, *w* = 38 %; Chem-Lab EV, Belgium) to assist plant digestion were purchased in ultra-pure quality. Ammonium nitrate (NH_4NO_3) solution (*c* = 1 mol L^{-1}) was prepared by weighing 80.04 g of NH_4NO_3 granulate (*w* = 98 %, Carl Roth GmbH, Germany) and filling volumetrically to one litre with ultra-pure water.

Filters for NH_4NO_3 soil extracts (Munktell Ahlstrom, Sweden) were of grade 1290 with a diameter of 150 mm and a weight per area of 84 g m^{-2}. All plastic consumables were cleaned by soaking in dilute HNO_3 (*w* = 3 %) for at least one day and subsequent thorough rinsing with ultra-pure water and drying in a laminar flow cabinet. Perfluoroalkoxy (PFA) vessels used for evaporation of Pb eluates after matrix separation were cleaned twice after each sample batch by HNO_3 vapour cleaning (Analab, Elemental Scientific, USA), rinsed with ultra-pure water and dried in a laminar flow cabinet. Cleaning of consumables, preparation of standards and ICP-MS analyses were performed in an ISO class 8 clean room. A BL224 BASIC analytical balance (XS instruments, Italy) with a readability of 0.01 g and a division of 0.0001 g was used for weighing.

Reagents and laboratory conditions for all work done at the University of Natural Resources and Life Sciences, Vienna (BOKU), are described elsewhere (Ziss et al., 2021).

2. Calibration standards and certified reference materials (CRMs)

Calibration standard solutions were prepared gravimetrically from the ICP multi-element standard solution VI (Merck Certipur, Darmstadt, Germany), in the following referred to as “MVI”. MVI contains 998 mg L^{-1} of Ca; 103 mg L^{-1} of Zn; 101 mg L^{-1} of Se; 99 mg L^{-1} of B and Be; 97 mg L^{-1} of As and Fe; 10.2 mg L^{-1} of Cd; 10.1 mg L^{-1} of U, 10.0 mg L^{-1} of Co, Li, Na, Mn, Ni, Sr, Te and V; 9.9 mg L^{-1} of Al, Bi, Cr, Cu, Ga, K, Mg, Mo, Pb and Tl, 9.8 mg L^{-1} of Ba and 9.7 mg L^{-1} of Ag and Rb. The stock solution was diluted to obtain an 11-point calibration ranging from (nominal) 0.005 to 50 ng g^{-1} Li. The specification of traceability of the respective elements can be found in **Table S1**.

Table S1: Specification of traceability for the elements contained in ICP multi-element solution VI (Merck Certipur)

Element	NIST Standard Reference Material
**Ag**	SRM 3151
**Al**	SRM 3101a
**As**	SRM 3103a
**B**	SRM 3107
**Ba**	SRM 3104a

Element	NIST Standard Reference Material
**Be**	SRM 3105a
**Bi**	SRM 3106
**Ca**	SRM 3109a
**Cd**	SRM 3108
**Co**	SRM 3113
**Cr**	SRM 3112a
**Cu**	SRM 3114
**Fe**	SRM 3126a
**Ga**	SRM 3119a
**K**	SRM 3141a
**Li**	SRM 3129a
**Mg**	SRM 3131a
**Mn**	SRM 3132
**Mo**	SRM 3134
**Na**	SRM 3152a
**Ni**	SRM 3136
**Pb**	SRM 3128
**Rb**	SRM 3145a
**Se**	SRM 3149
**Sr**	SRM 3153a
**Te**	SRM 3156
**Tl**	SRM 3158
**U**	SRM 3164
**V**	SRM 3165
**Zn**	SRM 3168a

For check of calibration and possible drift, a quality control (QC) solution was prepared by spiking a mixture of single-element standard solutions of Na 100 mg L^{-1} (Merck KGaA, Darmstadt, Germany), Mg 1000 mg L^{-1} (Merck KGaA, Darmstadt, Germany), Al 1001 ± 5 µg mL^{-1} (Inorganic Ventures, New Jersey, USA), K 1000 mg L^{-1} (Merck KGaA, Darmstadt, Germany), Ca 1000 mg L^{-1} (Merck KGaA, Darmstadt, Germany), Mn 999 ± 4 µg mL^{-1} (Inorganic Ventures, New Jersey, USA), Fe 1002 ± 4 µg mL^{-1} (Inorganic Ventures, New Jersey, USA), Zn 999 ± 4 µg mL^{-1} (Inorganic Ventures, New Jersey, USA), Se 1000 ± 3 µg mL^{-1} (CPI International, Santa Rosa, USA), Rb 1000 mg L^{-1}

(Merck KGaA, Darmstadt, Germany) and Ba 996 ± 4 µg mL^{-1} (Inorganic Ventures, New Jersey, USA) to an MVI calibration standard. The mass fractions contained in the QC solution are given in **Table S2**.

Table S2: Elemental mass fractions in the QC solution

Element	Mass fraction [ng g^{-1}]
**Cr, Ga, Mo, Ag, Tl, Pb**	2.8
**Li, V, Co, Ni, Cu, Cd, Te, Bi, U**	2.9
**Sr**	23
**Rb**	23
**As**	28
**Be, B**	29
**Al**	42
**Na**	43
**Ba**	52
**Zn**	59
**Mn**	72
**Se**	79
**K**	91
**Mg**	100
**Fe**	150
**Ca**	880

The plant CRMs used in this study were SRM1547 Peach Leaves (National Institute of Standards & Technology, USA), GBW10015 Spinach Leaves (National Research Centre for Certified Reference Materials, China) and ZC73008a Green Tea (NCS Testing Technology Co., China). For validation of soil analysis, ISE885 Brown Soil Pseudoclay (Wageningen Evaluating Programs for Analytical Laboratories, Netherlands) was used.

To assess the quality of matrix separation for Pb isotopic analysis, QC solutions ("QC SRM soil" and "QC SRM plants") simulating the matrix of the soil and plant samples were prepared based from the certified single-element isotopic reference standards SRM981 Common Lead Isotopic Standard (National Institute of Standards & Technology, USA) and SRM987 Strontium Carbonate (National Institute of Standards & Technology, USA) as well as ICP single-element standard solutions of Ca, Rb and Mg (1000 mg L^{-1} each, Merck, Germany) were used. The target elemental mass fractions are presented in **Table S3** and are based on the average mass fraction of Ca, Mg, Rb, Sr and Pb in the samples determined in multielement screening. For standard-sample bracketing (SSB) in Pb isotope ratio analysis, SRM981 was used.

Table S3: Elemental mass fractions in QC SRM soil and QC SRM plants

Element	QC SRM soil	QC SRM plant
**Mg [ng g^{-1}]**	-	30000
**Ca [ng g^{-1}]**	30000	65000
**Rb [ng g^{-1}]**	20	50
**Sr [ng g^{-1}]**	50	200
**Pb [ng g^{-1}]**	25	15

3. Multielement analysis

The instrumental parameters for the multielement analyses performed on the NexION 2000 (PerkinElmer, USA) are given in **Table S4**.

Table S4: Instrumental parameters for multielement analyses (NexION 2000)

Parameter	Feature
**Cell gas**	None
**Spray chamber temperature**	5 °C
**Interface cones**	Nickel
**Nebuliser**	PFA-ST-40 44296
**Nebuliser gas flow**	0.97-1.09 mL min^{-1}
**RF power**	1600 W
**Plasma gas flow**	16 L min^{-1}
**Auxiliary gas flow**	1.2-1.4 L min^{-1}
**Deflector voltage**	-14 V
**Data acquisition mode**	6 sweeps/reading, 1 reading/replicate, 6 replicates
**Dwell time per replicate**	50-150 ms
**Integration time**	150-900 ms
**RPa**	0-0.015 V
**RPq**	0.25 V
**Total time/sample**	60.508 s

4. Pb isotope ratio analysis

Details about the individual steps of the matrix separation method using the automated low-pressure chromatographic system prepFAST-MC (Elemental Scientific, USA) with a 3 ml bed-column filled with DGA resin (TrisKem International, Bruz, France) are given in **Table S5**.

Table S5: Matrix separation steps. *Load volume was dependent on the elemental mass fractions in the sample

Step	Volume	Flow rate	Reagent
**Condition column**	8 mL	2 mL min^{-1}	HNO_3 (c = 2 mol L^{-1})
**Load sample**	3-10 mL *	1 mL min^{-1}	HNO_3 (c = 2 mol L^{-1})
**Elute matrix**	6 mL	2 mL min^{-1}	HNO_3 (c = 2 mol L^{-1})
**Pre-elute Sr**	2 mL	1 mL min^{-1}	HNO_3 (c = 0.2 mol L^{-1})
**Elute Sr**	4 mL	1 mL min^{-1}	HNO_3 (c = 0.2 mol L^{-1})
**Elute Pb**	5 mL	1 mL min^{-1}	HNO_3 (c = 5 mol L^{-1})
**Wash column**	15 mL	1 mL min^{-1}	HCl (c = 0.1 mol L^{-1})

The instrumental parameters for the Pb isotope ratio analyses performed on the NU Plasma HR (NP048, Nu Instruments, UK) are given in **Table S6**.

Table S6: Instrumental parameters for Pb isotope ratio analyses (NU Plasma HR, NP048)

Parameter	Feature
**RF power**	1300 W
**Coolant flow**	13 L/min
**Auxiliary flow**	0.9 L/min
**Nebuliser pressure**	28 psi
**Interface cones**	Nickel, dry
**Sample introduction system**	Aridus II (Teledyne CETAC, USA) with MicroFlow PFA-ST nebulizer (ESI, USA)
**Measurement mode**	Static batch analysis (6 blocks with 10 measurement cycles with 10 s integration time)
**Resolution mode**	$m/\Delta m$ = ~300
**High voltage settings**	HV1: 3999.7 V; HV2: 3048.7 V; HV3: 2994.8 V; HV4: 1199.6 V; HV5: 2046.0 V; HV6: 1641.2 V
**Cup configuration**	H4: ^{208}Pb; H3: ^{207}Pb; H2: ^{206}Pb; H1: ^{205}Tl; Ax: ^{204}Pb; L1: ^{203}Tl; L2: ^{202}Hg

Parameter	Feature
**Axial mass / mass separation**	204 / 1
**Typical sensitivity**	385 V (µg g^{-1})

5. Data processing

5.1 Multielement analysis

Error propagation was conducted based on chapter 5 of the guide to the expression of uncertainties (GUM) (Joint Committee for Guides in Metrology, 2008). The calculated uncertainties were multiplied by 2 to obtain U (k = 2).

For the wash effect of elemental contents in washed versus unwashed lettuce leaves, the uncertainty was calculated according to equation (S1).

$$U_{\text{wash effect}} = \sqrt{\left(\frac{s_{\text{unwashed}}}{w_{\text{unwashed}}}\right)^2 + \left(\frac{s_{\text{washed}}}{w_{\text{washed}}}\right)^2} \quad \text{(S1)}$$

The error propagation for the estimation of elemental mass fractions in whole tomato fruits was performed according to equation (S2).

$$U_{\text{whole fruit}} = \sqrt{s_{\text{Pulp}}^2 + s_{\text{Skin}}^2 + s_{\text{Seeds}}^2} \quad \text{(S2)}$$

Analogously, the error propagation for the estimation of elemental mass fractions in unpeeled radish bulbs was performed according to equation (S3).

$$U_{\text{unpeeled}} = \sqrt{s_{\text{Bulb(peeled)}}^2 + s_{\text{Skin}}^2} \quad \text{(S3)}$$

z-scores were calculated based on DIN ISO 13528:2015 (E) (International Organization for Standardization, 2015) according to equation (S4) to check for agreement between certified values and the obtained data.

$$z = \frac{x_{\text{lab}} - X_{\text{ref}}}{s} \quad \text{(S4)}$$

x_{lab}: Mean result of this study
X_{ref}: Reference value
s: Standard deviation for proficiency testing, in this study: U given in the certificate

5.2 Pb isotope ratio analysis

For isotope ratios, calculation of U was modified based on the protocol of (Horsky et al., 2016). For mean isotope ratios, U_{average} was assessed using equation (S5) based on the s and U of the individual values.

$$U_{\text{average}} = \sqrt{(2s)^2 + U^2} \quad \text{(S5)}$$

For correction of IIF, two different calibration methods, namely external intra-elemental calibration using SSB and internal inter-elemental calibration using Tl, were used based on established approaches (Meija et al., 2012). IIF-correction of ^{207}Pb/^{206}Pb using SSB is shown in equation (S6).

$$(^{207}\mathrm{Pb}/^{206}\mathrm{Pb})_{\mathrm{corrected}} = \frac{(^{207}\mathrm{Pb}/^{206}\mathrm{Pb})_{\mathrm{sample,measured}}}{(^{207}\mathrm{Pb}/^{206}\mathrm{Pb})_{\mathrm{standard,\ mean}}} \times (^{207}\mathrm{Pb}/^{206}\mathrm{Pb})_{\mathrm{certified}} \quad \text{(S6)}$$

Equations (S7-S8) show how the isotope ratio $^{207}Pb/^{206}Pb$ was IIF-corrected using Tl as internal standard with a representative terrestrial natural Tl isotope ratio of $^{205}Tl/^{203}Tl_{natural}$ = 2.3875 (Belshaw et al., 1998).

$$(^{207}\mathrm{Pb}/^{206}\mathrm{Pb})_{\mathrm{corrected}} = (^{207}\mathrm{Pb}/^{206}\mathrm{Pb})_{\mathrm{measured}} \times \left(\frac{M(^{207}\mathrm{Pb})}{M(^{206}\mathrm{Pb})}\right)^{f} \quad \text{(S7)}$$

$$f = \ln\left(\frac{\left(\frac{^{205}\mathrm{Tl}}{^{203}\mathrm{Tl}}\right)_{\mathrm{natural}}}{\left(\frac{^{205}\mathrm{Tl}}{^{203}\mathrm{Tl}}\right)_{\mathrm{measured}}}\right) / \ln\left(\frac{M(^{205}\mathrm{Tl})}{M(^{203}\mathrm{Tl})}\right) \quad \text{(S8)}$$

References

Belshaw NS, Freedman PA, O'Nions RK, Frank M, Guo Y. A new variable dispersion double-focusing plasma mass spectrometer with performance illustrated for Pb isotopes. International Journal of Mass Spectrometry 1998; 181: 51-58.

Horsky M, Irrgeher J, Prohaska T. Evaluation strategies and uncertainty calculation of isotope amount ratios measured by MC-ICP-MS on the example of Sr. Analytical and Bioanalytical Chemistry 2016; 408: 351-367.

International Organization for Standardization. ISO 13528:2015; Statistical methods for use in proficiency testing by interlaboratory comparison, 2015, pp. 28.

Joint Committee for Guides in Metrology. Evaluation of measurement data - Guide to the expression of uncertainty in measurement (GUM), 2008.

Meija J, Yang L, Mester Z, Sturgeon RE. Correction of Instrumental Mass Discrimination for Isotope Ratio Determination with Multi-Collector Inductively Coupled Plasma Mass Spectrometry. In: Vanhaecke F, Degryse P, editors. Isotopic Analysis: Fundamentals and Applications using ICP-MS. WILEY-VCH, Weinheim, 2012, pp. 117-119.
